# Older Cancer Patients during the COVID-19 Epidemic: Practice Proposal of the International Geriatric Radiotherapy Group

**DOI:** 10.3390/cancers12051287

**Published:** 2020-05-19

**Authors:** Nam P. Nguyen, Vincent Vinh-Hung, Brigitta G. Baumert, Alice Zamagni, Meritxell Arenas, Micaela Motta, Pedro Carlos Lara, Arthur Sun Myint, Marta Bonet, Tiberiu Popescu, Te Vuong, Gokula Kumar Appalanaido, Lurdes Trigo, Ulf Karlsson, Juliette Thariat

**Affiliations:** 1 Department of Radiation Oncology, Howard University, Washington, DC 20060, USA; 2 Department of Radiation Oncology, University Hospital of Martinique, 97200 Fort-de-France, France; anhxang@gmail.com; 3 Institute of Radiation Oncology, Cantonal Hospital Graubuenden, 7000 Chur, Switzerland; brigitta.baumert@gmail.com; 4 Radiation Oncology Center, Department of Experimental, Diagnostic and Specialty Medicine, Sant’Orsola-Malpighi Hospital, University of Bologna, 40138 Bologna, Italy; alice.zamagni4@unibo.it; 5 Department of Radiation Oncology, Sant Joan de Reus University, University Rovira I Virgili, 43201 Tarragona, Spain; meritxell.arenas@gmail.com; 6 Department of Radiation Oncology, Hospital Papa Giovanni XXIII, 24127 Bergamo, Italy; Motta.micaela@libero.it; 7 Department of Radiation Oncology, Fernando Pessoa Canarias Las Palmas University, 35001 Las Palmas, Spain; pedrocarlos.lara@ulpgc.es; 8 Department of Radiation Oncology, Clatterbridge Cancer Center, Liverpool CH63 4JY, UK; sun.myint@nhs.net; 9 Department of Radiation Oncology, Arnau de Vilanova University Hospital, 25198 Lleida, Spain; mabonet.lleida.ics@gencat.cat; 10 Department of Radiation Oncology, Prof. Dr. Ion Chricuta Oncology Institute, 400015 Cluj-Napoca, Romania; tiberiu_popescu14@yahoo.com; 11 Department of Radiation Oncology, McGill University, Jewish General Hospital, Montreal, QC H3T1E2, Canada; te.vuong@mcgill.ca; 12 Department of Radiation Oncology, AMDI, Universiti Sains Malaysia, Penang 13200, Malaysia; gokudroid@gmail.com; 13 Department of Radiation Oncology, Instituto Portugues de Oncologia do Porto Francisco Martins Porto E.P.E, 4200-072 Porto, Portugal; lurdes.trigo@ipoporto.min-saude.pt; 14 Department of Radiation Oncology, International Geriatric Group, Washington, DC 20001, USA; karlssonulf30@gmail.com; 15 Department of Radiation Oncology, Baclesse Cancer Center, 14000 Caen, France; jthariat@gmail.com

**Keywords:** older, cancer patients, epidemic, corona virus 19, treatment

## Abstract

The coronavirus disease 19 (COVID-19) pandemic is unprecedented as it reached all countries in the world within a record short period of time. Even though COVID-19 infection may be just severe in any adults, older adults (65-year-old or older) may experience a higher mortality rate. Among those affected, cancer patients may have a worse outcome compared to the general population because of their depressed immune status. As the health resources of most countries are limited, clinicians may face painful decisions about which patients to save if they require artificial ventilation. Cancer patients, especially the older ones, may be denied supportive care because of their shorter life expectancy. Thus, special considerations should be taken to prevent infection of older cancer patients and to provide them with adequate social support during their cancer treatment. The following proposal was reached: (1) Education of health care providers about the special needs of older cancer patients and their risks of infection. (2) Special consideration such as surgical masks and separate scheduling should be made to protect them from being infected. (3) Social services such as patient navigators should be provided to ensure adequate medical supply, food, and daily transportation to cancer centers. (4) Close monitoring through phone calls, telecommunication to ensure social distancing and psychological support from patient family to prevent anxiety and depression. (5) Shorter course of radiotherapy by use of hypofractionation where possible to decrease the needs for daily transportation and exposure to infection. (6) Enrollment of older cancer patients in clinical trials for potential antiviral medications if infection does occur. (7) Home health care telemedicine may be an effective strategy for older cancer patients with COVID-19 infection to avoid hospital admission when health care resources become restricted. (8) For selected patients, immunotherapy and targeted therapy may become the systemic therapy of choice for older cancer patients and need to be tested in clinical trials.

## 1. Introduction

Coronavirus Disease 19 (COVID 19) is a pandemic of unprecedented epic proportion. From the first case officially reported in Wuhan in December 2019, it has spread rapidly to all countries in the world despite drastic government effort to contain it. To date, the number of patients infected has increased exponentially, causing a disproportional number of deaths among older patients (65 years-old or older) [1]. Even though COVID-19 infection may be just as severe in any adults, the mortality rates are higher among older adults and cancer patients [2]. As an illustration, Grasselli et al. [3] reported that among 1043 patients who were admitted to the ICU for severe respiratory distress, older patients defined as 64 year-old or older, mortality rate was significantly higher than younger patients. The mortality rate was, respectively, 36% and 15% for older and younger patients. Currently, there is no effective treatment for this disease. Until an effective vaccine is available which may take up to 18 months for its development, clinicians are faced with an ever-increasing number of patients and limited resources at their disposition. Because the number of ICU beds and ventilators is limited, physicians may face the painful choice about which patients may benefit from artificial ventilation and which ones to allow the disease to run its course. Those who are frail and over 80 years of age may not be the best candidates for artificial ventilation. As an illustration, in a previous study, older age was an independent factor for life-sustaining-treatment restriction [4]. Even though the data about COVID-19 are limited, the current evidence suggests that cancer patients may have a worse prognosis if infected [5]. In previous epidemics, among the ones who developed viral pneumonia of any etiology, a higher death rate was recorded in older cancer patients [6].

Thus, given the limited resources available, older cancer patients may not be eligible for artificial ventilation if they develop respiratory failure because of their shorter life expectancy, shortage of medical equipment, and limited recovering potential after a prolonged ICU stay. Management of older cancer patients during the current epidemic must take into consideration their frailty, decreased immunity, increased risk of infection, social isolation, and potential death if infection occurs. As an international organization devoted to the care of older cancer patients, women, and minorities, the International Geriatric Radiotherapy Group (http://www.igrg.org) [7] would like to develop practical recommendations during this time of uncertainty to guide clinicians on how to manage their older cancer patients based on preliminary data on COVID-19 and past viral epidemic studies such as the Severe Acute Respiratory Syndrome (SARS), Middle East Respiratory Syndrome (MERS), and Human Immunodeficiency Virus (HIV).

## 2. Staff Education during an Epidemic

At the beginning of an epidemic, it is of the utmost important to educate the staff about the pathogen infectivity, its mechanism of infection, the clinical syndrome associated with it, how to diagnose a potentially infected patient, and how to avoid infection when interacting with potential infected patients. As with any new pathogen, anxiety is the rule. In a study of 1216 health care workers (HCW) in Saudi Arabia during the MERS epidemic, knowledge about the disease transmission was poor, procedures to protect health care personnel were not followed properly, and compliance to hand hygiene and protective masks was low. Over 60% of HCW reported anxiety about getting infected [8]. It is understandable that as personnel on the front line, HCW are at high risk of being infected. They may transmit their disease to their family, older parents, or patients under their care who may die as a consequence of their exposure. There was a high rate of HCW infection at the beginning of the COVID-19 epidemic which was related to their lack of understanding of the virus mechanism of infection resulting in poor personal protection during prolonged exposure to the pathogen [9]. It has been reported that 3000 Chinese HCW were infected and at least 22 have died [10]. It is important to recognize that asymptomatic HCW can transmit disease [11]. Thus, it is vital to educate and protect HCW through emphasis that thorough disinfection of contaminated objects by viral droplets, proper use of personal protective equipment (PPE), and strict hand hygiene will minimize their risk of infection, significantly reduce their chance of infecting their patients and love ones, decrease their anxiety, and reduce the burden to their co-workers if they were to be infected.

Once HCW become comfortable with their role as providers during the epidemic, they should be educated about the special needs of older cancer patients to keep them safe.

It is often a challenge to reduce to a maximum the patient time spent in the cancer center to decrease their exposure to infection while recognizing that older cancer patients may require extra-time and attention due to their reduced mobility. The care for older cancer patients should be personalized based on their physical performance and socio-economic status. Patients who require radiotherapy treatment often present a challenge because of the need for daily treatment which may be extend to several weeks for curative intent.

In order to accommodate those older patients who are at high risk of dying if infected, special effort should be made to schedule them at a time convenient for their daily transportation while minimizing their interaction with other patients to reduce their infection exposure. For instance, they can come early in the morning before younger patients when the treatment room is likely less contaminated. If it is not feasible, they may stay in their vehicle and be contacted through cell phone to come when the treatment couch and their immobilization device are thoroughly disinfected. While waiting patients should be provided with a surgical mask while they stay in the car, and they should be kept at a safe distance from other patients if they are to stay in the waiting room. If they come with a caretaker, the latter should also wear a surgical mask because of the close and prolonged contact during the trip. As they may have limited mobility, which requires lifting from the wheelchair or stretcher, HCW should wear N-95 respiratory mask because of the close contact to their face. As asymptomatic infected HCW may transmit the virus to immunocompromised patients, port of N95 mask is mandatory when they are in close contact with those patients.

Patients should monitor their temperature at home or at the nursing home. Those who developed fever should not come for treatment and instead be referred to their primary care providers with advanced notice for further evaluation. Those precautions, even though cumbersome, are necessary because of the patient immunosuppression.

## 3. Special Consideration for Older Patients Who Require Radiotherapy for Cancer Treatment

Before starting radiotherapy, a frailty assessment such as the comprehensive geriatric assessment (CGA) should be completed to assess the patient clinical status and personalize treatment [12]. During treatment, patient clinical condition may change, which would require a modification of treatment strategy.

As an illustration, hypofractionation should be considered to reduce total treatment time and the need for daily transportation. Hypofractionation has been proven to be safe and effective in multiple randomized trials of various malignancies for both curative and palliative indications [13,14]. A single treatment to palliate pain from bony metastases may be selected for very frail patients. For others, accelerated hypofractionation may be an option. Another example is hypofractionation for a potential cure in older cancer patients with rectal cancer. Those patients could be re-assessed eight to twelve weeks after treatment. Patients with a complete response may be monitored. Those with a partial response may benefit from a boost with the Papillon technique or high dose rate brachytherapy (HDR) if available [15]. In patients with early stage non-small cell lung cancer, stereotactic body radiotherapy (SBRT) should be considered instead of conventional lobectomy as most older cancer patients presented with pre-existing comorbidity [16]. Lobectomy requires postoperative monitoring in the surgical intensive care unit (ICU) which is currently overwhelmed with infected patients who require artificial ventilation. In addition, more patients can be treated with a shortened schedule which further decreases the burden of a heavy workload for HCW. As treatment delay may allow the cancer to grow and/or metastasize, curative treatment should be initiated as soon as possible while ensuring the safety of both patients and HCW.

After treatment completion, telemedicine, if available, should be the ideal follow-up modality as it allows real time interaction between healthcare providers and their patients while respecting social distance. Current technologies such as Zoom allow direct interaction either through a land-connected phone or a smart phone if a computer is not available. Telemedicine has been demonstrated to reduce admission rate and the burden of traveling for patients who underwent surgery [17]. Patients will advanced-stage cancer also benefited from telemedicine as they underwent rehabilitation. Their pain and physical activity improved while they were monitored after hospital discharge [18]. An appointment or referral can be made if the patient needs to be seen in person. Other follow-up modalities such as a phone interview, although less desirable, could be considered for patient monitoring. It is important to emphasize that patients should not be perceived as having been abandoned by the medical community. Clinicians should indicate to the patient that those measures are designed to protect their safety and are temporary until an effective vaccine is available. Clear communication and transparency are needed to maintain patient trust of the medical system in times of uncertainty.

## 4. Special Consideration for Patients Who Require Surgery

Elective surgery such as breast cancer surgery may be cancelled or delayed because of the need to accommodate for COVID-19 patients who may require artificial ventilation. However, those measures are only temporary in anticipation of the peak of patient infection. Measures taken by public health officials such as social distancing, quarantine of infected patients and those exposed, stay-at-home order or lockdown, and wearing masks in public may slow down the rate of infection to allow the resumption of elective surgery. The hospital administration should take measures to ensure that both COVID-19 and non-COVID-19 patients are safe during their hospitalization. There is a need to separate COVID-19 and non-COVID-19 patient in separate wards and operating rooms. In addition, patients who undergo elective surgery should undergo COVID-19 testing to avoid inadvertent surgery for those who may be asymptomatic but infected which would put HCW at high risk of exposure during and following surgery [19]. Even though it is still controversial, polymerase chain reaction (PCR) of naso-, oro-pharyngeal secretions and sputum may provide fast and accurate testing [20]. However, as false negative tests are still possible, HCW should be provided with personal protective equipment (PPE) such as N-95 masks because of the risk of aerosolization of infective secretion during intubation [21].

## 5. Special Consideration for Older Cancer Patients

Anxiety and depression: Older cancer patients often developed anxiety, depression, and difficulty coping with their illness. Up to 25% of those patients may develop depression after cancer diagnosis [22]. Feelings of loneliness, and decreased cognitive function are frequently reported among older cancer patient compared to younger cancer patients or older patients without cancer [23,24]. In addition, limited access to food supply and fear of contracting the disease in public space may alter their nutrition and depress their immunity. Those psycho-social issues may further be exacerbated by social distancing which is now the norm to prevent their potential infection with asymptomatic family members. They need to be in touch with their friends and love one through contactless communications such as social media, telephone, face time, emails, text messages, or teleconference. If depression is diagnosed, a collaborative model involving primary care physicians, psychiatrists, therapists, psychologists, nurses, and social workers may be effective through telemedicine [25].

Transportation: During this pandemic, stay-at-home or lockdown orders have been issued by many governments across the globe to avoid disease spread. Those measures may affect transportation as violation may incur a fine. Before those emergency measures were installed, access to transportation was recognized as a barrier to treatment especially among older and minority patients with a limited income [26]. In a study of 406 patients with non-small cell lung cancer (NSCLC) with medical insurance, 29% were non-compliant with first-line chemotherapy. The lack of adequate transportation and older age were cited as major impediments in receiving chemotherapy, even among patients with a good performance status [27]. The issue is further compounded in older patients who have reduced mobility and require special mode of transportation to cater to their needs like a wheel chair or scooter [28]. As an illustration, among older adults who were physically active and lived in their own home, a lack of access to transportation lead to depression and health deterioration; additionally, depression became worse when they were unable to walk [29]. Thus, transportation is a key issue to provide adequate care to older cancer patients while remaining compliant to government directives.

Many studies in various countries have reported the efficacy of a patient navigation system to overcome the transportation barrier [30,31,32]. The patient navigator assessed many factors that can impact on patient care such as financial burden, medical insurance, fear, and disability through a psychosocial interview, arranged for the appropriate mode of transportation, and monitoring the patient to ensure that they received the appropriated medical care. A high rate of success up to 97% was reported among patients who frequently experienced discrimination because of their low socio-economic status [33]. During the HIV epidemic, patient navigation system was used successfully for HIV positive inmates to facilitate their integration back to the society after their release [33]. Thus, a patient navigation system should be an essential component of the medical response to the epidemic to avoid delay and treatment interruption among older cancer patients who may already have decreased cognitive function. All patients and their caretakers should receive a medical necessary certificate issued by their health care providers to come for treatment because of the lockdown or stay-at-home orders.

## 6. Special Consideration for Older Patients Who Become Infected during the Epidemic

Despite all the precautions taken, older cancer patients may become infected before or during the course of their treatment. Current guidelines mandate quarantine at home or in the nursing home until the patient become virus-free. If serious respiratory symptoms develop, they may require hospital admission. However, given the scarcity of hospital beds during the epidemic, older cancer patients may not be eligible for getting a ventilator if they develop respiratory failure. This is a cruel scenario that may arise when the number of patients infected rises significantly and overwhelms the health care system.

In this event, telemedicine may be the difference between life and death. There are currently many investigations to assess the efficacy of potential antiviral medications. From 23 January 2020 to 8 March 2020, there were 382 of those trials registered on the World Health Organization International Clinical Trials Registry Platform (ICTRP) and the list is increasing rapidly [34]. The current National Institute of Health (NIH) rules mandate recruitment of older cancer patients in any clinical trials in the US (http://www.nih.gov). Thus, clinicians should recruit older cancer patients in clinical antiviral trials if they get infected. However, since those experimental medications may have side effects, especially among older patient with many co-morbidity factors, close monitoring of those patients is mandatory during the trial. Telemedicine, if available, allows assessment of the patient clinical status in real time. Patient instructions and monitoring could be performed by a special unit at the hospital familiar with the study protocol after online consent. Vital signs such as temperature, respiratory rate, blood pressure, pulse oximetry, and patient movements can be monitored effectively through wearable devices [35]. Wearable technology combined with telemedicine has been investigated in cancer patients to improve their mental health, physical rehabilitation, and in the case of older cancer patients, treatment toxicity [36,37]. Quarantine instructions can be provided, and patient compliance monitored. Food and medicine supply could be delivered by people dressed with Protective Personnel Equipment (PPE). Oxygen can also be delivered at home to improve patient shortness of breath or those with hypoxia observed on pulse oxymetry. A metanalysis of telemedicine studies reported improved blood glucose control in diabetic patients compared to face-to-face medicine [38]. Thus, telemedicine may be effective as a mean to deliver home cancer care with infected older individuals. In addition, home care may reduce treatment cost and may potentially allow better quality of life because the patient is in a familiar environment instead of being isolated in a negative pressure room surrounded by complete strangers in full PPE [39]. In a previous community that acquired bacterial pneumonias, intravenous antibiotics administered at home were as effective as the ones given in the hospital setting [40]. As several medications currently being used experimentally against COVID-19, like hydroxychloroquine [41] and anti-HIV medications, are given by mouth, such approach is feasible in the home setting for older cancer patients. A baseline EKG is necessary for those taking antiviral medications because of their possible effect on the QT interval to avoid serious arrhythmia. With patient consent, their clinical status can be communicated to their family to ease their anxiety and in case of death immediate notification for funeral arrangements. If those elderly adapted telemedicine measures are not available, then other means of communication to monitor the patient clinical status during their treatment at home, such as text messages, email, telephone call and Skype may be used. Again, flexibility is the key to adapting to a changing environment.

## 7. Special Considerations for Immunotherapy and Targeted Therapy

### In Older Cancer Patients

Chemotherapy, when indicated, may further increased immunosuppression for older cancer patients with already compromised immunity, making them more sensitive to the virus and potential death. However, delaying chemotherapy may compromise the survival of those patients. This issue is further compounded by the shortage of diagnostic test kits to diagnose COVID-19 infection in asymptomatic patients. Inadvertent chemotherapy for those already immunocompromised and infected may lead to death. Thus, possible alternatives to avoid this scenario may be considered until testing becomes widely available. Immunotherapy with check point inhibitors has been proven effective for selective patients with various tumors such as lung cancer, either alone or in combination with chemotherapy for selective patients [42]. Among patients with selective biomarkers, immunotherapy is particularly attractive because of the lack of effect on the bone marrow and lower rates of treatment toxicity [43]. A meta-analysis of 13 randomized studies involving 6585 patients with locally advanced or metastatic NSCLC who underwent either immunotherapy (*n* = 3513) or chemotherapy (*n* = 3072) reported better tolerance to immunotherapy [44]. Even though bias is unavoidable in a meta-analysis, overall survival and progression-free survival were significantly increased for the immunotherapy group while adverse events such as anemia, neutropenia, and fatigue were significantly reduced. For patients with a poor performance status such as older cancer patients, preliminary evidence suggest that immunotherapy dose reduction may be as effective as a full dose for those with a poor performance status and more cost effective [45,46,47].

In addition, for HIV positive patients with cancer, immunotherapy may also enhance the effect of antiviral medications by decreasing the reservoir of viral laden T4 cells which are responsible for disease relapse following antiviral drugs cessation. Those reservoir T cells carry PD1 proteins which are the target of immunotherapy [48]. As COVID-19 and HIV share a similar structure, anti-HIV medications are now being used as experimental drugs against COVID-19 by the world health organization (http://www.who.int) [34,49]. If anti-HIV medications are effective against COVID-19, immunotherapy may have additional benefit for HIV-positive older cancer patients. Taken together, those preliminary studies suggest that immunotherapy may play a major role for selected older cancer patients and need to be investigated in future clinical trials.

Even though most trials only included a small number of older patients in the study, targeted therapy has also been reported to be effective and safe for selective patients with driver gene mutations [50]. As an illustration, for NSCLC with epidermal growth factor receptors (EGFR) mutations, gefinitib and erlotinib have been reported to be as effective among older patients as in younger patients with less side effects compared to conventional chemotherapy [51].

In an ideal world, all cancer patients should be tested for COVID-19 regardless of age. Our recommendations may also apply for younger cancer patients, but we have to take into consideration the economics of medicine with its constraints on the practice of medicine.

## 8. Conclusions

Management of older cancer patients requires education of HCW about their special need to prevent infection. Home health care with telemedicine for infected older cancer patients should be considered during COVID-19 epidemic as an alternative to hospital admission when health care resources become scarce. Infected patients should be enrolled in clinical trials for antiviral medications to assess their efficacy and toxicity. Immunotherapy and targeted therapy may play a role as a systemic therapy of choice for selected older cancer patients but need to be tested in clinical trials to test that hypothesis.
